# The Potent *In Vitro* Skin Permeation of Archaeosome Made from Lipids Extracted of *Sulfolobus acidocaldarius*

**DOI:** 10.1155/2013/782012

**Published:** 2013-12-26

**Authors:** Eskandar Moghimipour, Mohammad Kargar, Zahra Ramezani, Somayeh Handali

**Affiliations:** ^1^Nanotechnology Research Center, Ahvaz Jundishapur University of Medical Sciences, Ahvaz 61357-33184, Iran; ^2^Department of Microbiology, Jahrom Branch, Islamic Azad University, Jahrom, Iran

## Abstract

Archaeosomes are a new generation of liposomes that exhibit higher stabilities under different conditions, such as high temperatures, alkaline or acidic pH, and presence of bile salts in comparison with liposomes, and can be used in biotechnology including drug, gene, and vaccine delivery. The objective of this study was to prepare archaeosomes using lipid extracted from *Sulfolobus acidocaldarius* and evaluate their physicochemical properties. The lipids were extracted from *S. acidocaldarius* and assayed by High Performance Thin-Layer Chromatography (HPTLC). Archaeosomes were prepared using film method and methylene blue was used as drug model. They were characterized for their vesicle size and Differential Scanning Calorimetry (DSC) was used to investigate changes in their thermal behavior. The released amount of methylene blue was determined using a dialysis membrane and rat skin. HPTLC analysis of the extracted lipids showed that glycerol ether may be the major lipid with more than 78 percent probability. Results of particle size determination showed a mean size of 158.33 nm and the results of DSC indicated the possible interaction of methylene blue with lipids during the preparation of archaeosome. The addition of cholesterol significantly improved the encapsulation of methylene blue in the archaeosome so that the encapsulation efficiency was 61.66 ± 2.88%. The result of *in vitro* skin permeation showed that methylene blue could pass through skin model according to Peppas model and there was about 41.66% release after 6 h, whereas no release was observed through dialysis membrane. According to the results of the study, it is concluded that archaeosome may be successfully used as drug delivery system.

## 1. Introduction

Liposomes are colloidal particles with concentric phospholipid bilayers that are capable of encapsulating drugs [1]. Liposomes have several advantages including improvement of drug penetration into tissues, ability to entrap small molecules and macromolecules, reducing the toxicity of incorporated drugs, prolonging release of active pharmaceutical agents, protecting encapsulated agents from metabolic processes, biodegradability, and biocompatibility [2–7]. Despite these advances, a major limitation to the use of liposomes is their instability, high cost of production especially in large scales, and their relatively short half-life [5, 8]. Archaeosomes are a new generation of liposomes that are made from one or more polar ether lipids extracted from the archaea or synthetic archaeal lipids. These microorganisms live in unusual habitats including high salinity, low pH, high temperatures, and high pressures [9, 10] and have many biotechnological applications. Ether links are more stable against oxidation and high temperature than ester links [11]. Therefore, archaeosomes are more resistant to oxidation, chemical hydrolysis, bile salts, alkaline or acidic pH, and high temperatures [9, 10, 12]. Due to their extraordinary stability, which permits sterilization and filtration, archaeosomes have found many applications in vaccine and drug delivery [13]. *Sulfolobus acidocaldarius* is a thermoacidophilic archaea that lives at high temperature and acidic environments. The ability of growth under harsh conditions is related to the bipolar tetraether lipids in the plasma membrane [14]. This organism oxidizes sulfur and iron and has been employed in the industry for extracting metals [15]. In aqueous solution, the polar lipids extracted from *S. acidocaldarius* can form stable liposomes [16].

The aim of the study was to prepare archaeosomes using lipid extracted from *S. acidocaldarius* and evaluate factors that can affect their physicochemical properties.

## 2. Materials and Methods

Methylene blue, dipotassium phosphate dibasic (K_2_HPO_4_), magnesium sulfate heptahydrate (MgSO_4_·7H_2_O), ammonium sulfate ((NH_4_)_2_SO_4_), potassium chloride (KCl), calcium nitrate tetrahydrate (Ca(NO_3_)_2_·4H_2_O), sulfur, cholesterol, chloroform, and methanol were purchased from Merck, Germany. Sephadex G-25 and yeast extract were obtained from Sigma, Germany, and QUELAB, Canada, respectively. Sulfuric acid was provided by UNI-CHEM, Germany.* Sulfolobus acidocaldarius* was kindly donated by the National Iranian copper Industries Co. Sarcheshmeh, Kerman, Iran.

### 2.1. Extraction of Lipids from *S. acidocaldarius*

*S. acidocaldarius* cells were grown in 9K-medium, which contained (gram per liter): (NH_4_)_2_SO_4_ (3), K_2_HPO_4_ (0.5), MgSO_4_·7H_2_O (0.5), Ca(NO_3_)_2_·4H_2_O (0.01), and KCl (0.1). Sulfur (10 gram per liter) and 0.1% yeast extract were added to the basal medium and adjusted to pH 1.7 using sulfuric acid. Cultures were incubated in rotary shakers (IKA KS4000i, Germany) for 7 days at 70°C. Cultures were filtered for removing the sulfur and then sulfur-free cells were lyophilized. Extraction of lipids from lyophilized cells was carried out by stirring with chloroform-methanol (2 : 1, v/v) for 1 h at room temperature. The suspension was passed through a sintered glass filter, and the residue reextracted for an additional hour. Combined filtrates were evaporated, taken up in chloroform-methanol-water (60 : 30 : 4.5, v/v/v), and passed through Sephadex G-25 for removal of nonlipid contaminations [15].

### 2.2. High Performance Thin-Layer Chromatograph (HPTLC)

HPTLC was performed on glass bucked silica gel 60 F 254 (Merck) plates of 10 × 10 cm with the help of Camag Linomat-IV applicator (E. Merck KGaA). All plates were first activated by heating in 150°C for 30 min. Different developing solvents including chloroform-methanol-water (65 : 25 : 4, v/v/v), chloroform, diethyl ether (9 : 1, v/v), and chloroform-methanol-water (60 : 10 : 1, v/v/v) were used [15]. A 25 *μ*L sample of lipids was spotted on the plates with a Hamilton syringe and chromatography was performed.

### 2.3. Preparation of Archaeosomes

In the beginning *λ*max of methylene blue was determined and its calibration curve was drawn. Archaeosomes were prepared from the extracted lipid using thin film method. Briefly, 1% methylene blue was added to the lipids solution that was extracted from *S. acidocaldarius* and then the mixture was evaporated in a rotary evaporator (Heidolph, Germany). When the thin film was formed in the round-bottom flask, it was hydrated with phosphate buffer. The suspension was agitated by vortex for 30 min and then sonicated for 45 min [2, 3]. Also, formulations containing 20 mg cholesterol in combination with the above mentioned materials were prepared.

### 2.4. Measurement of Liposome Size

The average diameters of archaeosomes were determined using a particle sizer Qudix, ScatterO Scope I system (Korea) at 25°C before and after homogenization [17]. Homogenization was used for reducing the particle size of archaeosomes. 10 mL of samples was homogenized (Ultra Turrax IKA T-25, Germany) at 2000 rpm at 25°C for 30 min.

### 2.5. Differential Scanning Calorimetry (DSC)

The calorimetric analysis was performed in order to determine the properties of lipids previously structured in the archaeosomes and the effect of methylene blue on the thermograms was also evaluated. The DSC curves were recorded using a DSC-1 Mettler Toledo oven with a temperature range of 0 to 200°C for 6 min [17].

### 2.6. Evaluation of the Loading Efficacy

The archaeosomal suspension was centrifuged at 20000 rpm for 15 min (VS-35SMTI, Korea), and the supernatant was analyzed at 660 nm using a spectrophotometer (Biochrom WAP Biowave II).

### 2.7. *In Vitro* Drug Release Studies

*In vitro* methylene blue release from the archaeosomes was determined using dialysis membrane method and a specially designed Franz diffusion cell. Samples were put in a dialysis membrane (BETAGEN, width 40 mm). The receptor chamber contained 22 mL distilled water and was continually stirred using a magnet stirrer at 37°C. An aliquot of 3 mL of sample was withdrawn from each batch at definite time intervals (1, 2, 3, 4, 5, and 6 h) and replaced with the same amount of distilled water to maintain sink condition. Then the concentration of released methylene blue was monitored using a UV spectrophotometer at 660 nm [17].

### 2.8. *In Vitro* Skin Permeation

Male Wistar rat skin was used as model membrane. The skin was hydrated by immersion in water for 24 h before experiment. Then it was mounted on the Franz-type diffusion cells with the stratum corneum side facing upward into the donor compartment. The donor compartment was filled with methylene blue containing archaeosomes. An aliquot of 3 mL of sample was withdrawn from the receptor compartment at 1, 2, 3, 4, 5, and 6 h and replaced with the same volume of distilled water at 37°C to maintain the volume constant. The amount of methylene blue in the receptor phase was assayed using formerly mentioned UV spectrophotometer apparatus at 660 nm [18]. The study was carried out in accordance with the guideline and permission of the Animal Ethics Committee of Jundishapur University of Medical Sciences, Ahvaz, Iran.

### 2.9. Drug Release Kinetics

The release mechanism was evaluated using different kinetic models including zero-order, first-order, Higuchi, and Korsmeyer-Peppas. The data were reported as mean ± SD and frequently as percents.

## 3. Results 

100 mg lipid was extracted from each 2 gram cell and total lipids were determined by HPTLC analysis. HPTLC is an advanced form of thin layer chromatography (TLC) that can analyze mixtures by separating the compounds and determining the number of components in a mixture.

**Figure 1:**
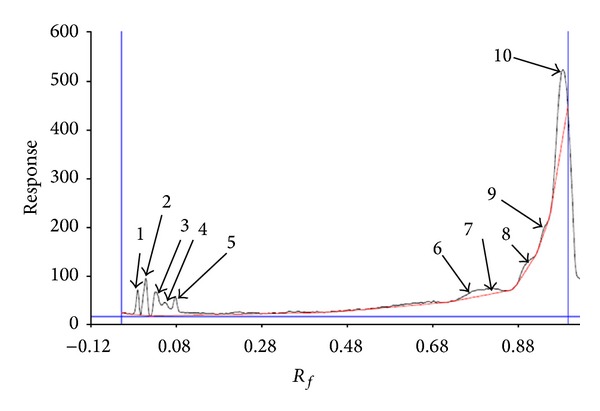
Chromatography of lipids in solvent system containing chloroform-methanol-water (65 : 25 : 4, v/v/v) scanned at 254 nm using Camag HPTLC scanner.

**Figure 2:**
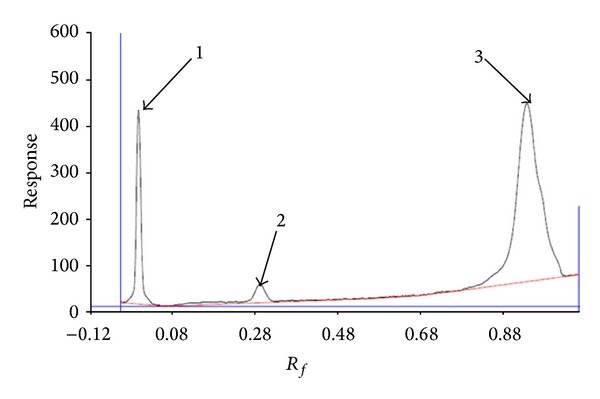
Chromatography of lipids in solvent system containing chloroform, diethyl ether (9 : 1, v/v) scanned at 254 nm using Camag HPTLC scanner.

**Figure 3:**
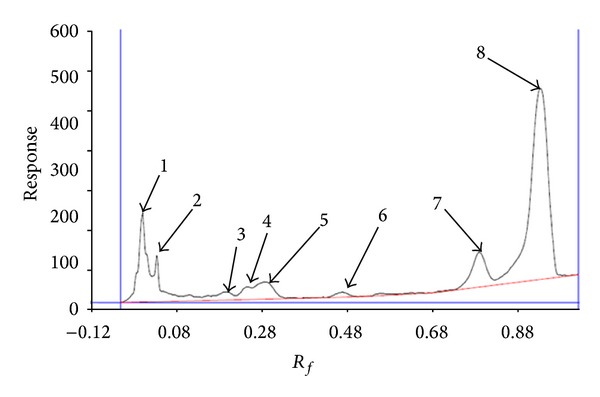
Chromatography of lipids in solvent system containing chloroform-methanol-water (60 : 10 : 1, v/v/v) scanned at 254 nm using camag HPTLC scanner.

As shown in Figures 1, 2, and 3, ten, three, and eight spots were detected at 254 nm on the plate with solvent system containing chloroform-methanol-water (65 : 25 : 4, v/v/v), chloroform, diethyl ether (9 : 1, v/v), and chloroform-methanol-water (60 : 10 : 1, v/v/v), respectively.

**Figure 4:**
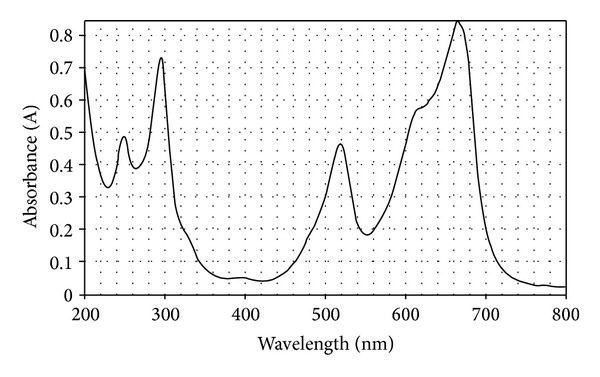
UV spectra of methylene blue.

A UV spectrum of methylene blue was shown in Figure 4 and its *λ*max was 660 nm.

**Figure 5:**
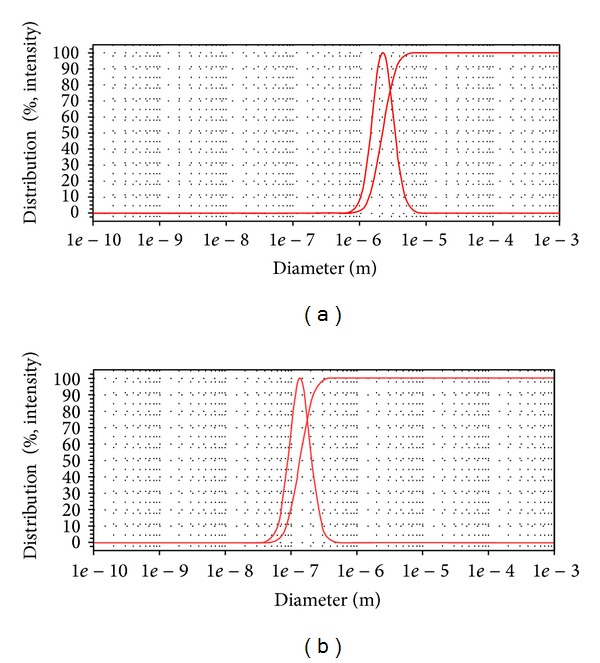
Particle size graphs of methylene blue containing archaeosomes (a) before homogenization and (b) after homogenization (30 min).

The average size of archaeosomes before and after homogenization was 1980 and 158.33 nm, respectively (Figures 5(a) and 5(b)). According to the results, homogenization decreased the particle size of archaeosomes.

**Figure 6:**
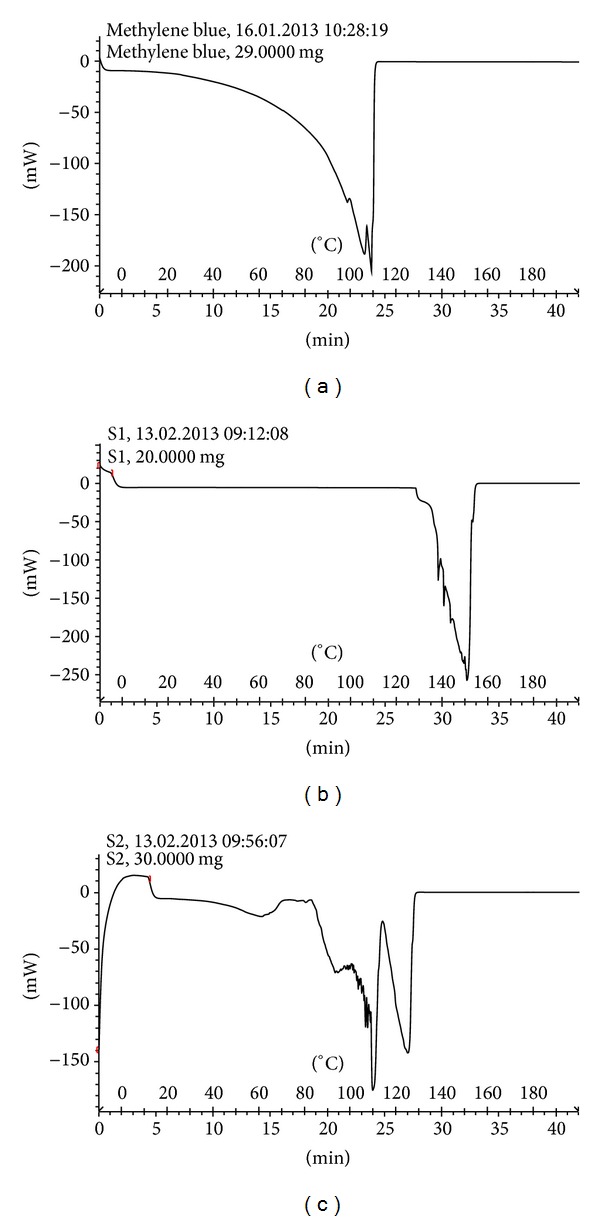
(a) Differential Scanning Calorimetry spectra of methylene blue (b) Archaeosomes without methylene blue and (c) Methylene blue containing archaeosomes.

DSC results for methylene blue, archaeosomes without methylene blue, and methylene blue containing archaeosomes are shown in Figures 6(a), 6(b), and 6(c), respectively. From comparing the melting point of methylene blue, archaeosomes without methylene blue and methylene blue containing archaeosomes, it can be concluded that there is a possible interaction of methylene blue with lipids during the preparation of archaeosome.

The encapsulation efficiency of methylene blue in formulations without cholesterol was less than 7%, but by adding cholesterol, 61.66 ± 2.88% of the methylene blue was encapsulated in archaeosomes.

The results of release study by Franz diffusion cell and cellulose membrane showed no transport into receptor phase, while the result of *in vitro* skin permeation showed that methylene blue could pass through skin model and the value of release from archaeosome in 1, 2, 3, 4, 5, and 6 h was 10.83 ± 0.00%, 16.66 ± 0.00%, 20.83 ± 5.83%, 31.66 ± 0.75%, 40.83 ± 5.58, and 41.66 ± 0.75%, respectively, while no release was observed from dialysis membrane. The result of *in vitro* skin permeation showed that methylene blue could pass through skin model according to Peppas model.

## 4. Discussion

Archaeosomes are a new generation of liposomes that are prepared from natural archaeal membrane lipids and have potential application in drug and vaccine delivery. According to Figure 1, two components at *R*_*f*_: 0.00 and *R*_*f*_: 0.00 could not move far in this solvent system. They were, respectively, about 4.94% and 10.56% of the total. The third at *R*_*f*_: 0.03 with 10.81%, the forth at *R*_*f*_: 0.06 with 5.63%, the fifth at *R*_*f*_: 0.08 with 8.01%, the sixth at *R*_*f*_: 0.79 with 8.22%, the seventh at *R*_*f*_: 0.81 with 5.29%, the eighth at *R*_*f*_: 0.90 with 4.61%, the ninth at *R*_*f*_: 0.94 with 1.67%, and the tenth at *R*_*f*_: 0.99 with 40.17% of the total were observed. Minnikin et al. in 1971 employed this solvent system for detecting lipids in the bacterial membranes. The results indicated the presence of three major phospholipids including phosphatidylglycerol, diphosphatidylglycerol, and phosphatidylethanolamine [19]. In addition, this solvent system was successfully employed by Langworthy et al. in 1977 and Fager et al. in 1977 for separation of lysophosphatidylethanolamine and phosphatidylcholine, and for determination of glycolipids and acidic lipids [15, 20]. It is believed that the points 3–10 may be evidences for the presence of phosphatidylcholine, lysophosphatidylethanolamine, glycolipids, or acidic lipid. However, still some components were not eluted from the spotting point. In order to check the presence of other lipids, another HPTLC plate was developed with solvent system mixture of chloroform, diethyl ether (9 : 1, v/v). As shown in Figure 2, the first component was at *R*_*f*_: 0.00, meaning that this solvent system was not suitable for detecting this component. It is about 17.35% of the total. The second component was at *R*_*f*_: 0.26 with 3.77% and the third at *R*_*f*_: 0.95 with 78.88% of total. This solvent system has been used by Langworthy in 1977 for detection of lipids containing glycerol ethers. It is suggested that the second and third components may be the lipid containing glycerol ethers [15]. Another HPTLC plate was developed with solvent system containing chloroform-methanol-water (60 : 10 : 1, v/v/v). As illustrated from Figure 3, eight spots were detected at 254 nm on this solvent system. The first component at *R*_*f*_: 0.01 with 13.32%, the second at *R*_*f*_: 0.04 with 5.94%, the third at *R*_*f*_: 0.21 with 1.59%, the forth at *R*_*f*_: 0.26 with 2.35%, the fifth at *R*_*f*_: 0.31 with 5.57%, the sixth at *R*_*f*_: 0.50 with 1.15%, the seventh at *R*_*f*_: 0.83 with 9.20%, and the eighth at *R*_*f*_: 0.98 with 60.88% of total were observed. This solvent system was used by Langworthy in 1977 for separation of lipids containing glycerol ethers [15]. According to their results, spots 2–8 may be lipids composed of glycerol ethers.

As shown in Figures 5(a) and 5(b), homogenization is an important factor in the archaeosomes preparation, and their size control. It is believed that homogenization probably prevents the aggregation and fusion of particles. Reduction in the particle size is important parameter for improving the performance of poorly soluble drugs [21]. Also, the results of a study demonstrated that homogenization time can significantly affect the particle size of vesicles [22]. It has been accepted that decreasing the particle size of vesicles may cause an increase in the penetration of encapsulated drugs into the deeper skin layers [23].

DSC is a great tool which can be used to investigate the interaction between liposomes and drug molecules [24]. By comparing the shift in the peak the drug of melting point, carrier without drug and carrier with drug, it can be realized that whether or not the drug is loaded in the carrier. The DSC curves of methylene blue showed peaks in the region 95–110°C, corresponding to the melting point of the compound. The DSC curve of archaeosomes without methylene blue showed endothermic peaks in the regions 135–150°C (Figure 6(b)) relating to its phase transfer, while the DSC curve of methylene blue containing archaeosomes showed endothermic peaks at 90–125°C (Figure 6(c)). The shift in the endothermic peak of archaeosomes indicated that methylene blue was successfully encapsulated in the archaeosomes and could interact with the bilayer structure and change its thermal behavior.

According to the results, the encapsulation efficiency of the archaeosomes was significantly influenced by the presence of cholesterol. It is suggested that cholesterol may increase the stability and modify the fluidity of archaeosomes prepared from lipid extracted by archaea. Cholesterol has been employed as helper lipid in the liposome formulation to improve further stability, rigidity and decrease leakage of the encapsulated drugs [25, 26]. Also, it has been widely used to modify drug release, improve physical stability, and prolong circulation half-life of liposomal drug* in vivo *[24]. Cholesterol interacts with fatty acids of liposomes via hydrogen binding, enhancing the cohesiveness and mechanical strength of the membrane [21]. Gonzalez et al., in 2009, used total lipids of *Halorubrum tebenquichens* for production of archaeosome asa new source of adjuvancy and for entrapment of bovine serum albumin. According to their results, the encapsulation efficiency was approximately 34% and the mean size of archaeosomes was 564 ± 22 nm. Their results also revealed that archaeosomes prepared with total polar lipid from the archea could be successfully used as vaccine delivery system [27]. Barbeau et al. in 2011 prepared and evaluated archaeosomes based on synthetic archaeal tetraether lipid and compared them with conventional liposomes. They used carboxyfluorescein as a drug model. The results showed that 70% of the encapsulated carboxyfluorescein was lost within 3 h, while a significant improvement in stability was observed with archaeosome, which released only 20% at the same time. They coated the archaeosome with a polyethylene glycol (PEG) in order to achieve a stabilizing nanovector and demonstrated that small proportions of PEGylated archaeolipid added to liposomal formulations increased stability and allowed slow release of the encapsulated dye [9].

Regarding the results, archaeosome may enhance permeation through the skin. It seems that increased permeability of the methylene blue by archaeosome may be related to the composition of their lipids that are similar to stratum corneum lipids, which are likely to enter stratum corneum lipids more readily, to fuse with endogenous lipids, and may act as penetration enhancers. Skin has been used as an application site of therapeutic drugs to avoid the hepatic first pass effect and side effects in the gastric intestinal tract [28]. For penetration of drugs across the skin, the main barrier is stratum corneum [29]. Liposomes penetrate the stratum corneum, by adhering onto the surface of the skin, loosening the lipid structure of the stratum corneum, promoting impaired barrier function of this layer to the drug and subsequent increase skin partitioning of the drugs [1, 30, 31]. Also, the results revealed that the methylene blue was released from archaeosome according to Peppas model (RSQ = 0.994 and MPE = 28.23%). Peppas model is dependent on the fraction of drug released at time, rate constant, and release exponent [32]. The physicochemical properties and the nature of drug and absorption enhancers are very important factors in enhancing skin permeation of the drugs [18]. Methylene blue is a polar compound and the stratum corneum layer of skin is the restriction barrier for transdermal delivery of polar compounds. It is believed that follicular shunt route is responsible for the permeation of polar molecules and drugs. Nevertheless, it is accepted that these routes comprise a fractional area for permeation of approximately 0.1% of total permeation. Consequently, penetration enhancement techniques have been concentrated on increasing transport across the stratum corneum rather than via the appendages [30]. According to our findings, archaeosome can be employed as penetration enhancer for polar compounds and drugs.

Many studies indicated that archeal lipids are good sources for preparation of liposomes due to their remarkable thermostability and safety. They can be employed in several biotechnological applications as delivery systems for genes, cancer imaging agents, and drugs [33]. Due to their phagocytosis by macrophages, conventional liposomes have been only applied as antigen carriers and adjuvant [34]. Krishnan et al. in 2001 studied the adjuvant activity of archaeosome that was prepared from lipid extracted from *Methanobrevibacter smithii*. They reported that archaeosomes could show adjuvant activity and induced T helper and cytotoxic T lymphocyte (CTL) responses to entrapped antigen [35]. Also, it has been previously reported that archaeosomes can induce a strong response and sustain antigen specific cytotoxic T lymphocyte (CTL) to their encapsulated peptide or protein [36].

Additional work in this way is suggested to trace the archaeosome in cancer cells for evaluating the potential application in delivery of molecules. Ultimately, it is aimed to exploit a nanodrug delivery system made from lipid of archaea that can be used for treatment of different diseases.

## 5. Conclusion

It is concluded that it is possible to prepare archaeosomes from archaeal lipids. According to the results, the encapsulation efficiency was found to be higher when the cholesterol was added and loading of methylene blue in archaeosomes exhibited release properties; thus, archaeosome may be used as carrier for drug delivery and used for treatment of different diseases.

## References

[1] Patel HJ, Trivedi DG, Bhandari AK, Shah DA (2011). Penetration enhancers for transdermal drug delivery system: a review. *Journal of Pharmaceutics and Cosmetology*.

[2] Chetanachan P, Akarachalanon P, Worawirunwong D (2008). Ultrastructural characterization of liposomes using transmission electron microscope. *Advanced Materials Research*.

[3] Mohammadi Samani S, Montaseri H, Jamshidnejad M (2009). Preparation and evaluation of cyproterone acetate liposome for topical drug delivery. *Iranian Journal of Pharmaceutical Sciences*.

[4] Trommer H, Neubert RHH (2005). Screening for new antioxidative compounds for topical administration using skin lipid model systems. *Journal of Pharmacy and Pharmaceutical Sciences*.

[5] Moghimipour E, Handali S (2013). Liposomes as drug delivery systems: properties and applications. *Research Journal of Pharmaceutical, Biological and Chemical Sciences*.

[6] Dua JS, Rana AC, Bhandari AK (2012). Liposome: methods of preparation and applications. *International Journal of Pharmaceutical Sciences and Research*.

[7] Di Paolo D, Pastorino F, Brignole C (2008). Drug delivery systems: application of liposomal anti-tumor agents to neuroectodermal cancer treatment. *Tumori*.

[8] Jain S, Khomane K, Jain AK, Dani P (2011). Nanocarriers for transmucosal vaccine delivery. *Current Nanoscience*.

[9] Barbeau J, Cammas-Marion S, Auvray P, Benvegnu T (2011). Preparation and characterization of stealth archaeosomes based on synthetic PEGylated archaeal tetraether lipid. *Journal of Drug Delivery*.

[10] Benvegnu T, Lemiègre L, Cammas-Marion S (2008). Archaeal lipids: innovative materials for biotechnological applications. *European Journal of Organic Chemistry*.

[11] Van de Vossenberg JLCM, Driessen AJM, Konings WN (1998). The essence of being extremophilic: the role of the unique archaeal membrane lipids. *Extremophiles*.

[12] Khosravi-Darani K, Pardakhty A, Honarpisheh H, Rao VSNM, Mozafari MR (2007). The role of high-resolution imaging in the evaluation of nanosystems for bioactive encapsulation and targeted nanotherapy. *Micron*.

[13] Kanichay R, Boni LT, Cooke PH, Khan TK, Chong PL-G (2003). Calcium-induced aggregation of archaeal bipolar tetraether liposomes derived from the thermoacidophilic archaeon *Sulfolobus acidocaldarius*. *Archaea*.

[14] Bagatolli L, Gratton E, Khan TK, Chong PL-G (2000). Two-photon fluorescence microscopy studies of bipolar tetraether giant liposomes from thermoacidophilic archaebacteria *Sulfolobus acidocaldarius*. *Biophysical Journal*.

[15] Langworthy TA (1977). Comparative lipid composition of heterotrophically and autotrophically grown *Sulfolobus acidocaldarius*. *Journal of Bacteriology*.

[16] Khan TK, Chong PL-G (2000). Studies of archaebacterial bipolar tetraether liposomes by perylene fluorescence. *Biophysical Journal*.

[17] Moghimipour E, Ramezani Z, Handali S (2013). Solid lipid nanoparticles as a delivery System for *Zataria multiflora* essential oil: formulation and characterization. *Current Drug Delivery*.

[18] Shokri J, Azarmi S, Fasihi Z, Hallaj-Nezhadi V, Nokhodchi A, Javadzadeh Y (2012). Effects of various penetration enhancers on percutaneous absorption of piroxicam from emulgels. *Research in Pharmaceutical Sciences*.

[19] Minnikin DE, Abdolrahimzadeh H, Baddiley J (1971). The interrelation of phosphatidylethanolamine and glycosyl diglycerides in bacterial membranes. *Biochemical Journal*.

[20] Fager RS, Shapiro S, Litman BJ (1977). A large-scale purification of phosphatidylethanolamine, lysophosphatidylethanolamine, and phosphatidylcholine by high performance liquid chromatography: a partial resolution of molecular species. *Journal of Lipid Research*.

[21] Luo Y, Chen D, Ren L, Zhao X, Qin J (2006). Solid lipid nanoparticles for enhancing vinpocetine’s oral bioavailability. *Journal of Controlled Release*.

[22] Moghimipour E, Aghel N, Mahmoudabadi Z, Ramezani Z, Handali S (2012). Preparation and characterization of liposomes containing essential oil of *Eucalyptus camaldulensis* leaf. *Jundishapur Journal of Natural Pharmaceutical Products*.

[23] Chen Y, Wu Q, Zhang Z, Yuan L, Liu X, Zhou L (2012). Preparation of curcumin-loaded liposomes and evaluation of their skin permeation and pharmacodynamics. *Molecules*.

[24] Tabbakhian M, Rogers JA (2012). Interaction of insulin, cholesterol-derivatized mannan, and carboxymethyl chitin with liposomes: a differential scanning calorimetry study. *Research in Pharmaceutical Sciences*.

[25] Samad A, Sultana Y, Aqil M (2007). Liposomal drug delivery systems: an update review. *Current Drug Delivery*.

[26] Tseng L-P, Liang H-J, Chung T-W, Huang Y-Y, Liu D-Z (2007). Liposomes incorporated with cholesterol for drug release triggered by magnetic field. *Journal of Medical and Biological Engineering*.

[27] Gonzalez RO, Higa LH, Cutrullis RA (2009). Archaeosomes made of Halorubrum tebenquichense total polar lipids: a new source of adjuvancy. *BMC Biotechnology*.

[28] Pathan IB, Setty CM (2009). Chemical penetration enhancers for transdermal drug delivery systems. *Tropical Journal of Pharmaceutical Research*.

[29] Mbah CJ, Uzor PF, Omeje EO (2011). Perspectives on transdermal drug delivery. *Journal of Chemical and Pharmaceutical Research*.

[30] Benson HAE (2005). Transdermal drug delivery: penetration enhancement techniques. *Current Drug Delivery*.

[31] Gonzlez-Paredes A, Manconi M, Caddeo C, Ramos-Cormenzana A, Monteoliva-Snchez M, Fadda AM (2010). Archaeosomes as carriers for topical delivery of betamethasone dipropionate: in vitro skin permeation study. *Journal of Liposome Research*.

[32] Dash S, Murthy PN, Nath L, Chowdhury P (2010). Kinetic modeling on drug release from controlled drug delivery systems. *Acta Poloniae Pharmaceutica*.

[33] Schiraldi C, Giuliano M, De Rosa M (2002). Perspectives on biotechnological applications of archaea. *Archaea*.

[34] Higa LH, Schilrreff P, Perez AP (2012). Ultradeformable archaeosomes as new topical adjuvants. *Nanomedicine*.

[35] Krishnan L, Sad S, Patel GB, Sprott GD (2001). The potent adjuvant activity of archaeosomes correlates to the recruitment and activation of macrophages and dendritic cells in vivo. *Journal of Immunology*.

[36] Benvegnu T, Lemiègre L, Cammas-Marion S (2009). New generation of liposomes called archaeosomes based on natural or synthetic archaeal lipids as innovative formulations for drug delivery. *Recent Patents on Drug Delivery and Formulation*.

